# Grading Diabetic Retinopathy Using Comparative Assessment: A Pilot Study Comparing Paired Image Comparisons With Direct Grading

**DOI:** 10.7759/cureus.102559

**Published:** 2026-01-29

**Authors:** Mohammed Al-Roubaie

**Affiliations:** 1 Department of Eye and Vision Sciences, University of Liverpool, Liverpool, GBR

**Keywords:** blind image quality assessment, confusion matrix, deep learning, diabetic retinopathy, fundus photography, image grading, mcnemar test, paired comparisons, tele-ophthalmology screening

## Abstract

Introduction

Accurate grading of diabetic retinopathy is essential for effective screening, clinical decision-making, and evaluation of automated diagnostic systems. Conventional grading relies on categorical severity scales, which are subject to inter- and intra-observer variability, particularly among less-experienced or junior graders and in cases with subtle disease features. Comparative assessment using paired image comparisons may offer a complementary approach by reframing grading as a relative severity judgement and potentially reducing grading variability.

Methods

This pilot study evaluated retinal fundus photographs obtained from a publicly available dataset. Ninety images spanning the spectrum of diabetic retinopathy severity were graded using two approaches: direct grading according to the International Clinical Diabetic Retinopathy Severity Scale and comparative assessment using paired image comparisons. Both methods were performed twice by a junior clinician following structured training to assess repeatability. Classification performance for discrimination between the presence and absence of diabetic retinopathy was compared using confusion matrices and McNemar’s test.

Results

Comparative assessment demonstrated higher overall accuracy and improved specificity compared with direct grading across repeated grading rounds, while maintaining high sensitivity. Paired image comparison showed greater consistency between grading attempts, whereas direct grading exhibited greater variability. Differences in classification performance between methods were statistically significant.

Conclusion

In this pilot study, comparative assessment using paired image comparisons outperformed conventional direct grading for discrimination between the presence and absence of diabetic retinopathy when applied by a junior grader. These findings suggest that relative severity judgement may represent a viable alternative or adjunct to traditional categorical grading systems, particularly in contexts where grading variability is a concern. Larger studies involving multiple graders and real-world screening images are required to validate these findings and define the clinical role of comparative assessment.

## Introduction

Diabetic retinopathy (DR) remains a leading cause of visual impairment among working-age adults worldwide and represents a major public health challenge despite advances in screening and treatment programmes [1-3]. Accurate identification and grading of DR severity are essential for determining referral pathways, monitoring disease progression, and guiding timely intervention [1,4]. Globally, DR is estimated to affect over 100 million people and remains a leading cause of visual impairment among working-age adults [2].

The International Clinical Diabetic Retinopathy Severity Scale (ICDRSS) is the most widely adopted framework for categorising DR severity in both clinical practice and research [1]. While this system provides a standardised approach, its application requires graders to integrate multiple retinal features into discrete ordinal categories, a process that is inherently subjective and vulnerable to inter- and intra-observer variability [4-6]. This variability is particularly pronounced among less experienced graders and in cases with subtle or borderline retinal changes [6-8].

Grading variability is particularly pronounced among less-experienced or junior graders, who may have limited exposure to the full spectrum of DR severity and may find absolute categorisation challenging when features are subtle or borderline. This variability has important implications for screening programmes and research studies that rely on human grading as a reference standard, as inconsistent classification can lead to unnecessary referrals, delayed treatment, or biased performance estimates for automated systems. Consequently, there is a need for alternative grading approaches that reduce cognitive load, improve consistency, and may be more robust when applied by junior or less-experienced graders.

Limitations in grading reliability have important implications for both clinical screening programmes and the development of automated diagnostic systems. Studies evaluating human graders and machine-learning algorithms have highlighted the absence of a true gold standard for DR grading and the impact of grader disagreement on performance assessment [6,9]. These challenges have prompted interest in alternative approaches to image evaluation that may reduce cognitive load and improve consistency.

Comparative assessment using paired image comparisons offers a fundamentally different strategy for image evaluation. Rather than assigning absolute severity categories, observers are asked to judge which of two images demonstrates greater disease severity. By shifting the task from absolute categorisation to relative judgement, comparative assessment may mitigate grading variability, particularly among junior or less-experienced graders, by simplifying decision-making and reducing reliance on precise threshold definitions. Paired comparison methods have a long theoretical foundation in psychophysics and visual perception research and have been shown to produce reliable ordinal rankings in situations where absolute classification is difficult [10-12]. This approach may be particularly well suited to retinal image analysis, where disease features often exist along a continuum rather than within discrete thresholds.

The aim of this pilot study was to compare the performance of direct grading using the ICDRSS with comparative assessment using paired image comparisons for binary discrimination between the presence and absence of DR. By evaluating repeatability and classification accuracy across both methods, this study seeks to explore whether comparative assessment may represent a viable alternative or adjunct to conventional DR grading approaches.

## Materials and methods

Study design and image dataset

This was a retrospective pilot study using publicly available, de-identified retinal fundus photographs obtained from the Kaggle Diabetic Retinopathy Detection dataset curated by EyePACS [13]. As all images were anonymised and freely accessible, formal ethical approval and informed consent were not required.

A total of 90 fundus photographs were selected to represent the full spectrum of DR severity. Images were distributed across five severity categories according to the ICDRSS: no DR, mild non-proliferative DR (NPDR), moderate NPDR, severe NPDR, and proliferative DR [1].

Observer training

Grading was performed by a single junior clinician with formal medical training and clinical exposure to ophthalmology. Prior to image assessment, the clinician underwent structured training provided by a senior ophthalmologist at the Royal Liverpool University Hospital, Liverpool, United Kingdom. Training focused on recognition of key DR features and application of the ICDRSS criteria [1].

Grading was performed by a single trained observer, and no inter-observer comparisons were undertaken in this pilot study. The observer completed a separate practice set of fundus photographs, not included in the analysis, to familiarise themself with lesion recognition and severity thresholds. During study grading, the observer was blinded to dataset labels, prior grading decisions, and the image order was randomised for each grading round. To assess repeatability, both direct grading and paired image comparison tasks were performed twice.

Direct grading

All 90 fundus photographs were independently graded according to the ICDRSS [1]. Each category was also assigned a label: Normal images with absent DR were labelled ‘0’, images containing mild NPDR were labelled ‘1’, images containing moderate NPDR were labelled ‘2’, images containing severe NPDR were labelled ‘3’, and images containing proliferative DR were labelled ‘4’, giving a total of five grading groups. Direct grading was performed twice, separated by the paired comparison task, to assess grading consistency.

Paired comparisons

Comparative assessment was performed using paired image comparisons, in which the observer was required to select the image demonstrating greater DR severity within each pair [10,11]. A total of 3,000 unique paired comparisons were generated from the same image set using a predefined randomisation script. Each pair comprised two images drawn from different ICDRSS severity categories, ensuring that no pair contained images from the same grade and thereby allowing valid relative severity judgements in the absence of a ground-truth ordering within categories. Images were presented side-by-side, and the observer selected the image perceived to represent more advanced disease.

Each image appeared multiple times across different pairings to ensure broad coverage of inter-grade comparisons. The paired comparison task was repeated twice to assess repeatability. Relative image rankings were derived from aggregated pairwise comparison outcomes using established paired comparison and comparative judgement principles [10-12].

Outcome measures

Grading performance was evaluated for discrimination between the presence of DR (ICDRSS grades 1-4) and the absence of DR (grade 0). This binary outcome was selected to reflect clinically relevant screening decisions and to allow direct comparison between grading approaches used in DR screening programmes, where referral decisions are typically based on disease presence versus absence [3,14].

Statistical analysis

Classification performance was summarised using confusion matrices. Diagnostic performance was evaluated using sensitivity, specificity, and accuracy derived from confusion matrices, with 95% confidence intervals (CIs) calculated using the Wilson score method. Between-method comparisons were performed using McNemar’s test for paired binary outcomes, with exact two-tailed p-values calculated using the binomial distribution [15]. Statistical significance was defined as a p-value less than 0.05.

## Results

Image dataset and grading categories

A total of 90 retinal fundus photographs were included in the analysis. Images were distributed across five DR severity categories according to the ICDRSS, comprising 10 images with no DR, 10 with mild NPDR, 30 with moderate NPDR, 30 with severe NPDR, and 10 with proliferative DR. The distribution of images across severity categories is summarised in Table 1.

**Table 1:** Distribution of retinal fundus images by diabetic retinopathy severity category. Diabetic retinopathy severity was classified according to the ICDRSS [2]. NPDR: non-proliferative diabetic retinopathy; PDR: proliferative diabetic retinopathy; ICDRSS: International Clinical Diabetic Retinopathy Severity Scale

Diabetic Retinopathy	Label	Number of images
Normal	0	10
Mild NPDR	1	10
Moderate NPDR	2	30
Severe NPDR	3	30
PDR	4	10

Paired image comparison: round 1

The first round of paired image comparison assessed the observer’s ability to discriminate between the presence and absence of DR by directly comparing pairs of fundus photographs from different severity groups. Classification outcomes for paired image comparison in Round 1 are summarised using a confusion matrix in Table 2.

**Table 2:** Confusion matrix for paired image comparison (Round 1) Confusion matrices show binary classification performance for discrimination between the presence (ICDRSS grades 1–4) and absence (ICDRSS grade 0) of diabetic retinopathy. Values represent the number of retinal fundus photographs classified in each category. ICDRSS: International Clinical Diabetic Retinopathy Severity Scale; DR: diabetic retinopathy

Prediction	Ground truth: DR present	Ground truth: DR absent
Predicted DR present	80	2
Predicted DR absent	0	8

Direct grading: round 1

Direct grading was performed independently of paired comparisons, with images assessed individually and assigned a DR severity grade. For the purpose of performance comparison, results were dichotomised into DR present or absent. Classification outcomes for direct grading in Round 1 are presented in Table 3.

**Table 3:** Confusion matrix for direct grading (Round 1) Confusion matrix showing binary classification performance for direct grading in Round 1. DR presence was defined as ICDRSS grades 1–4 and absence as ICDRSS grade 0. Values represent the number of retinal fundus photographs classified in each category. ICDRSS: International Clinical Diabetic Retinopathy Severity Scale; DR: diabetic retinopathy

Prediction	Ground truth: DR present	Ground truth: DR absent
Predicted DR present	80	10
Predicted DR absent	0	0

Paired image comparison: round 2

Following completion of the first direct grading and paired comparison rounds, the paired image comparison task was repeated to assess repeatability. The confusion matrix for paired image comparison in Round 2 is shown in Table 4.

**Table 4:** Confusion matrix for paired image comparison (Round 2) Confusion matrix showing binary classification performance for paired image comparison in Round 2. DR presence was defined as ICDRSS grades 1–4 and absence as ICDRSS grade 0. Values represent the number of retinal fundus photographs classified in each category. ICDRSS: International Clinical Diabetic Retinopathy Severity Scale; DR: diabetic retinopathy

Prediction	Ground truth: DR present	Ground truth: DR absent
Predicted DR present	80	0
Predicted DR absent	0	10

Direct grading: Round 2

Direct grading was also repeated to assess intra-observer consistency. As with Round 1, results were dichotomised into DR present or absent for comparison. Classification outcomes for direct grading in Round 2 are summarised in Table 5.

**Table 5:** Confusion matrix for direct grading (Round 2) Confusion matrix showing binary classification performance for direct grading in Round 2. DR presence was defined as ICDRSS grades 1–4 and absence as ICDRSS grade 0. Values represent the number of retinal fundus photographs classified in each category. ICDRSS: International Clinical Diabetic Retinopathy Severity Scale; DR: diabetic retinopathy

Prediction	Ground truth: DR present	Ground truth: DR absent
Predicted DR present	80	10
Predicted DR absent	0	0

Diagnostic performance metrics

Diagnostic performance metrics derived from the confusion matrices are summarised in Table 6. Paired image comparison demonstrated superior specificity and overall accuracy compared with direct grading across both rounds, while maintaining high sensitivity. Specifically, specificity improved markedly with paired image comparison, increasing from 0% with direct grading in both rounds to 80% in Round 1 and 100% in Round 2 of paired comparison; 95% CIs for sensitivity, specificity, and accuracy were calculated using the Wilson score method.

**Table 6:** Diagnostic performance of direct grading and paired image comparison Sensitivity, specificity, and accuracy were calculated using standard definitions derived from confusion matrix outcomes for binary classification of DR presence (ICDRSS grades 1–4) versus absence (ICDRSS grade 0); 95% CIs were calculated using the Wilson score method. ICDRSS: International Clinical Diabetic Retinopathy Severity Scale; DR: diabetic retinopathy

Method	Sensitivity % (95% CI)	Specificity % (95% CI)	Accuracy % (95% CI)
Paired image comparison – Round 1	100.0 (95.4–100.0)	80.0 (49.0–94.3)	97.8 (92.3–99.4)
Direct grading – Round 1	100.0 (95.4–100.0)	0.0 (0.0–27.8)	88.9 (80.7–93.9)
Paired image comparison – Round 2	100.0 (95.4–100.0)	100.0 (72.2–100.0)	100.0 (95.9–100.0)
Direct grading – Round 2	100.0 (95.4–100.0)	0.0 (0.0–27.8)	88.9 (80.7–93.9)

Between-method comparison using McNemar's test

McNemar’s test was used to compare paired binary classification outcomes between grading methods across the same set of 90 images. There was no statistically significant difference between the two rounds of direct grading (Round 1 vs Round 2; p = 1.000), nor between the two rounds of paired image comparison (Round 1 vs Round 2; p = 0.500), indicating good within-method repeatability.

In contrast, statistically significant differences were observed when comparing direct grading with paired image comparison. Paired image comparison demonstrated superior classification performance compared with direct grading in all between-method comparisons, including Round 1 direct grading versus Round 1 paired comparison (p = 0.008), Round 1 direct grading versus Round 2 paired comparison (p = 0.002), Round 2 direct grading versus Round 1 paired comparison (p = 0.008), and Round 2 direct grading versus Round 2 paired comparison (p = 0.002). The results of McNemar’s test, including exact p-values, are presented in Table 7.

**Table 7:** McNemar’s test comparing grading methods for binary diabetic retinopathy classification. McNemar’s test was used to compare paired binary classification outcomes between direct grading and paired image comparison methods across the same set of 90 retinal fundus photographs. Exact two-tailed p-values were calculated using the binomial distribution. Statistical significance was defined as p < 0.05.

Comparison	N	Test	Exact p-value
Direct grading Round 1 vs Direct grading Round 2	90	McNemar (exact)	1.000
Direct grading Round 1 vs Paired comparison Round 1	90	McNemar (exact)	0.008
Direct grading Round 1 vs Paired comparison Round 2	90	McNemar (exact)	0.002
Direct grading Round 2 vs Paired comparison Round 1	90	McNemar (exact)	0.008
Direct grading Round 2 vs Paired comparison Round 2	90	McNemar (exact)	0.002
Paired comparison Round 1 vs Paired comparison Round 2	90	McNemar (exact)	0.500

## Discussion

In this pilot study, comparative assessment using paired image comparisons demonstrated superior performance compared with conventional direct grading for binary discrimination between the presence and absence of DR. Across both grading rounds, paired image comparison showed improved specificity for identifying images without DR and greater consistency between repeated assessments, while maintaining high sensitivity. In contrast, direct grading was associated with a tendency toward over-classification of disease in images without DR.

A central motivation for this study was the well-recognised variability in DR grading, particularly at the threshold between disease absence and early disease. Accurate identification at this stage is critical for screening programmes and clinical decision-making [2-4]. Conventional categorical grading systems require graders to integrate multiple retinal features into discrete severity levels defined by international grading frameworks [1], a process that is inherently subjective and vulnerable to inter- and intra-observer variability. This variability is especially pronounced among less-experienced or junior graders and in cases with subtle or borderline disease features, with important implications for both clinical screening and algorithm development [6].

Comparative assessment addresses this challenge by reframing grading as a fundamentally different cognitive task. Rather than assigning absolute severity categories, observers make relative judgements between image pairs, reducing reliance on fixed threshold definitions. Paired comparison methods have a long-standing theoretical foundation in psychophysics and visual perception research and have been shown to produce reliable ordinal rankings in situations where absolute classification is challenging [10-12]. Similar comparative approaches have been explored in other areas of medical image interpretation and ophthalmic imaging, supporting their external validity as tools for complex visual assessment. The improved specificity and repeatability observed in this study suggest that relative severity judgements may be particularly well suited to retinal image analysis, where pathological features often exist along a continuum rather than within discrete thresholds.

An important finding of this study was the improved repeatability of paired image comparison across grading rounds. Direct grading demonstrated greater variability between repeated assessments, whereas paired image comparison remained stable. In the context of DR screening programmes, where reproducibility across time and between graders is essential to ensure safe patient triage, this characteristic may represent a clinically meaningful advantage, particularly when grading is performed by junior clinicians or in high-throughput screening environments [3,4].

These findings also have implications for scalability and future integration into automated diagnostic pipelines. Human grading is frequently used as a reference standard for training and validating machine learning models for DR detection, despite known limitations in grading consistency [6,9]. Comparative assessment may offer an alternative framework for generating more robust ordinal rankings of disease severity, which could complement existing grading paradigms in multi-grader screening workflows and algorithm development, potentially improving the reliability of reference standards.

This study has several limitations. It was conducted using a relatively small image set and a single junior clinician as the observer, which limits generalisability. The small number of DR-absent images (n = 10) restricts the precision of specificity estimates and contributes to wider CIs, and findings relating to specificity should therefore be interpreted cautiously. Only binary classification of DR presence versus absence was evaluated, and performance across finer severity distinctions was not assessed. In addition, images were sourced from a publicly available dataset [13] and may not fully reflect the variability in image quality, artefacts, and clinical context encountered in real-world screening programmes.

Despite these limitations, this pilot study provides preliminary evidence that comparative assessment using paired image comparisons may outperform conventional direct grading for identifying DR, particularly in settings where grading variability is a concern. Larger studies incorporating multiple graders, expanded datasets, and real-world screening images are required to validate these findings and to define the role of comparative assessment as an adjunct or alternative to traditional grading systems in clinical practice.

## Conclusions

In this pilot study, comparative assessment using paired image comparisons demonstrated superior performance to conventional direct grading for binary discrimination between the presence and absence of DR, particularly when applied by a junior grader. Paired image comparison showed improved specificity for identifying images without DR and greater consistency across repeated grading rounds, while maintaining high sensitivity. In contrast, direct grading was associated with a tendency toward over-classification of disease in images without DR.

These findings suggest that comparative assessment may offer a robust and reproducible alternative to traditional categorical grading approaches, particularly in contexts where subtle retinal features make absolute severity classification challenging. By reducing cognitive load and emphasising relative severity judgements, paired image comparison may enhance grading reliability in both clinical and research settings. While limited by its single-observer design and modest dataset size, this study provides preliminary evidence supporting the potential role of comparative assessment in DR evaluation. Further research incorporating multiple graders, larger datasets, and real-world screening images is warranted to define its utility alongside established grading systems and emerging automated diagnostic tools.
